# De Pneumonia Typhode sive Nervosa; adnexis hujus morbi historiis

**Published:** 1800-03

**Authors:** 


*De Pneumonia Typhode ſive Nervoſa; adnexis hujus morbi hiſtoriis. Auctore* M. C. G. CAPPEL, M. D. 8vo. pp. 216. 2s. 6d. 1799. Göttingen. London, *Geiſweiller.*

In an ingenious preface, the author apologizes for having undertaken to write on a particular ſpecies of pneumonia, while it is ſuppoſed that every thing relative to that diſeaſe has been duly conſidered by ſo great a number of ancient and modern writers. He therefore adviſes thoſe who are of this opinion, to reflect upon the diverſified principles laid down by authors, reſpecting that diſorder, and to decide after a careful compariſon, whether the different ſpecies of it are ſufficiently diſtinguiſhed, whether the diagnoſis of each writer is properly explained, and whether the method of cure itſelf is duly inveſtigated. Dr. Cappel, without heſitation, anſwers theſe queſtions in the negative; and he endeavours to prove the neglectful conduct of phyſicians, in this reſpect, by the great diverſity of opinion concerning ſeveral of the remedies; ſo that ſome conſider venæſection indiſpenſably neceſſary, while others abſolutely reject it; and others again fluctuate between the two extremes. This cenſure is farther juſtified, by the uncommon praiſe beſtowed upon one and the ſame remedy, in every ſpecies of pneumonia.* Hence the author is induced to believe that, notwithſtanding the many excellent writings on this ſubject, neither the different nature of the diſeaſe, nor the method of cure has been accurately ſtated. And as it is one of the moſt difficult and dangerous maladies which very frequently afflict and prove fatal to mankind, it appeared to him deſerving the inquiry of medical men, eſpecially for the following reaſons.

The ſpecies of pneumonia are frequently ſo difficult to be diſtinguiſhed, that even the moſt ſagacious phyſician cannot with certainty determine the preciſe nature of the diſeaſe. In the next place, the practitioner to whom the cure of it is intruſted, cannot delay his active meaſures for ſeveral days, ſo as to abſtract the method of cure from time and circumſtances; but his duty requires the immediate choice and application of remedies againſt ſo dreadful an attack. Laſtly, the phyſician has here an opportunity of diſplaying, in a very eminent degree, his practical talent; as there are few diſeaſes, the event of which, whether favourable or fatal, depends ſo much on his advice and ingenuity, as that of pneumonia.

Induced

---

* Noſologi plures pneumoniæ ſpecies enumerant, attamen vel in omnibus venæſectionem commendant. Conf. B. DE SAUVAGES, *Noſologia Methodica.* Lips. 1791. Tom. II. p. 286. et ſeqq.

Induced by these considerations, the author did not think the present inquiry either useless or superfluous; he submits therefore his work to the equitable judgment of the public, while he observes, it will perhaps be thought presumptuous, that a young man destitute of experience, the great guide of medical practice, has ventured to write upon this practical subject. But as he has, for nearly a whole year, frequented the lectures of the illustrious Frank, in the Clinical Institut at Vienna, he thence derived much useful information, and especially from the Clinical Wards of the celebrated Dr. Nord, first physician to the Great Civil Infirmary, in the same city, where cases of pneumonia daily occurred: besides these valuable sources, he has also perused the best writers on the subject.

In the introduction, the author chiefly dwells upon the manner of distinguishing, and ascertaining the inflammatory symptoms accompanying pneumonia; while he is of opinion, that in fever combined with inflammation, this should first be attended to; and that there ought to be admitted as many kinds of inflammation, as there are general of fevers.—He next examines the various divisions of fever proposed by Doctors Selle, Frank, Hufeland, Reil and Brown; which last he considers as the *best*, and adds a few observations on the origin of fevers.

The work itself does not admit of abridgment; it is written in a perspicuous and manly style, though the reasonings frequently betray an eager and inexperienced mind.—Among the most useful external remedies, the author reckons warm cataplasms, tepid baths of the feet, and the whole body, volatile liniments, tepid vapours, sinapisms and blisters: to the list of internal remedies he has placed the aqua ammoniæ acetatæ, polygala senega, Kerme's mineral, emetics, camphor, musk, mercury, Peruvian bark, and opium.

From the annexed *six* histories of pneumonic cases we learn, that four of the patients were cured, one dismissed incurable, and only *one* died. It deserves to be remarked, that all these cases occurred under the direction of the celebrated Franks, father and son, who seldom prescribe venæsection in pneumonia, and *never* in that of the typhoid, or nervous kind.

We seriously recommend to our readers the perusal of this essay

